# Self-Assembling Peptide-Based Magnetogels for the Removal of Heavy Metals from Water

**DOI:** 10.3390/gels9080621

**Published:** 2023-08-01

**Authors:** Farid Hajareh Haghighi, Roya Binaymotlagh, Laura Chronopoulou, Sara Cerra, Andrea Giacomo Marrani, Francesco Amato, Cleofe Palocci, Ilaria Fratoddi

**Affiliations:** 1 Department of Chemistry, Sapienza University of Rome, Piazzale Aldo Moro 5, 00185 Rome, Italy; farid.hajarehhaghighi@uniroma1.it (F.H.H.); roya.binaymotlagh@uniroma1.it (R.B.); sara.cerra@uniroma1.it (S.C.); andrea.marrani@uniroma1.it (A.G.M.); francesco.amato@uniroma1.it (F.A.); ilaria.fratoddi@uniroma1.it (I.F.); 2 Research Center for Applied Sciences to the Safeguard of Environment and Cultural Heritage (CIABC), Sapienza University of Rome, Piazzale Aldo Moro 5, 00185 Rome, Italy

**Keywords:** magnetogels, magnetic nanoparticles, peptide-based hydrogels, hydrogel composites, water purification, Cr(III), Co(II), Ni(II)

## Abstract

In this study, we present the synthesis of a novel peptide-based magnetogel obtained through the encapsulation of γ-Fe_2_O_3_-polyacrylic acid (PAA) nanoparticles (γ-Fe_2_O_3_NPs) into a hydrogel matrix, used for enhancing the ability of the hydrogel to remove Cr(III), Co(II), and Ni(II) pollutants from water. Fmoc-Phe (*Fluorenylmethoxycarbonyl*-Phenylalanine) and diphenylalanine (Phe_2_) were used as starting reagents for the hydrogelator (Fmoc-Phe_3_) synthesis via an enzymatic method. The PAA-coated magnetic nanoparticles were synthesized in a separate step, using the co-precipitation method, and encapsulated into the peptide-based hydrogel. The resulting organic/inorganic hybrid system (γ-Fe_2_O_3_NPs-peptide) was characterized with different techniques, including FT-IR, Raman, UV-Vis, DLS, ζ-potential, XPS, FESEM-EDS, swelling ability tests, and rheology. Regarding the application in heavy metals removal from aqueous solutions, the behavior of the obtained magnetogel was compared to its precursors and the effect of the magnetic field was assessed. Four different systems were studied for the separation of heavy metal ions from aqueous solutions, including (1) γ-Fe_2_O_3_NPs stabilized with PAA, (γ-Fe_2_O_3_NPs); (2) Fmoc-Phe_3_ hydrogel (HG); (3) γ-Fe_2_O_3_NPs embedded in peptide magnetogel (γ-Fe_2_O_3_NPs@HG); and (4) γ-Fe_2_O_3_NPs@HG in the presence of an external magnetic field. To quantify the removal efficiency of these four model systems, the UV-Vis technique was employed as a fast, cheap, and versatile method. The results demonstrate that both Fmoc-Phe_3_ hydrogel and γ-Fe_2_O_3_NPs peptide magnetogel can efficiently remove all the tested pollutants from water. Interestingly, due to the presence of magnetic γ-Fe_2_O_3_NPs inside the hydrogel, the removal efficiency can be enhanced by applying an external magnetic field. The proposed magnetogel represents a smart multifunctional nanosystem with improved absorption efficiency and synergic effect upon applying an external magnetic field. These results are promising for potential environmental applications of γ-Fe_2_O_3_NPs-peptide magnetogels to the removal of pollutants from aqueous media.

## 1. Introduction

Worldwide, due to the increase in industrialization levels, the protection of the environment from industrial wastes has become more and more important over the past decades. The presence of toxic metal ions in industrial wastewaters can result in adverse effects on both human health and the environment even at low concentrations because these inorganic species are generally nondegradable in nature [1,2,3]. Some of the most common metal pollutants are cadmium (Cd), lead (Pb), mercury (Hg), nickel (Ni), arsenic (As), copper (Cu), chromium (Cr), cobalt (Co), and zinc (Zn). Table 1 summarizes the primary industrial sources, health side effects and the permitted quantity of these heavy metals, based on recent literature data [4,5,6,7,8].

In the past decades, there have been admirable efforts to develop fast and efficient techniques to remove metal ions from aqueous solutions, and different approaches have been studied and developed for water remediation, including ion exchange, electrochemical treatments, membrane separation, and flocculation [1,9]. However, these methods often suffer from their own limitations in terms of complexity, cost, and efficiency. In recent years, sol- and gel-based adsorption strategies have attracted increasing attention for scaling-up the removal process because of their simplicity, high efficiency, and low-cost operation. To this aim, polymers, clay minerals, and carbon-based materials have been investigated as adsorbents, and promising results have been obtained in this regard [10,11,12,13,14]. On this basis, an ideal adsorbent should have high adsorption capacity, fast adsorption kinetics, good chemical stability, and easy preparation method [2].

Hydrogels have been recently introduced as suitable alternatives as absorbent materials due to their highly porous structure and adsorbing functional groups, as well as their large surface area and high swelling ability [15,16,17,18,19,20,21]. They are colloidal materials that possess a three-dimensional (3D) network based on amphipathic polymer building blocks. The polymers link to each other, forming an insoluble 3D matrix that can absorb and entrap a significant amount of water [22,23]. For water treatment applications, it is highly recommended to use biocompatible absorbents, and among them, peptide-based hydrogels for example can be suitable candidates [1,15]. In general, hydrogels can absorb large amounts of metal ions inside their matrices, due to the presence of suitable functional groups. As is well-known, on this basis, hydrogels physically or chemically interact with pollutants via one or more mechanisms. One of the major mechanisms of heavy metals removal by hydrogels is based on electrostatic interactions that usually occur when, as a function of pH, hydroxyl and carboxyl groups of hydrogels are deprotonated, imparting negative charges to the hydrogel. Other possible interactions involve H-bond formation. These types of hydrogels can be easily used and scaled up for industrial applications of wastewater treatment because of their cost-effectiveness and standard synthetic methods. In particular, they can be prepared by using an enzymatic approach and under mild conditions, which are highly suitable for large-scale production [19,24].

To enhance hydrogels adsorption efficiency and reduce their operational costs, they can be hybridized (or combined) with magnetic nanoparticles (NPs) to form “magnetogels”, which are promising organic-inorganic nanohybrids for environmental and biological applications, as they combine benefits of both hydrogels and magnetic nanoparticles into a single inorganic/organic hybrid [25,26,27,28,29,30,31,32]. In fact, magnetic sorbents entrapped within hydrogel-based materials can promote heavy metals removal thanks to the large number of functional groups on hydrogel surfaces, thus improving adsorption selectivity as well as sorbent capacity. These magnetogels are considered as soft smart multifunctional nanosystems, which provide the possibility for enhancing the adsorption efficiency of hydrogels upon applying an external magnetic field. In addition, the presence of magnetic nanoparticles can modify hydrogels’ structure through covalent or non-covalent interactions, which offers the possibility to finely tune their physico-chemical properties. The porous magnetogels present in their structure active functional groups (e.g., carboxyl, hydroxyl and amino groups) that are able to remove contaminants by means of electrostatic interactions, ionic exchange, or complexation with contaminants such as heavy metal ions. More importantly, the interaction of magnetogels with an external magnetic field can promote the separation, collection, and reuse of hydrogel adsorbents and also have an enhancing effect on the adsorption of magnetogels [28]. Among the various types of magnetic NPs, maghemite (γ-Fe_2_O_3_) and magnetite (Fe_3_O_4_) nanoparticles have been employed for biological and environmental applications due to their high magnetization, biocompatibility, and well-assessed synthesis methods [25]. These two types of magnetic nanoparticles both exhibit strong on/off superparamagnetic properties in the presence/absence of a magnetic field, respectively. Although maghemite NPs (γ-Fe_2_O_3_NPs) show a slightly smaller magnetic moment compared to magnetite NPs (Fe_3_O_4_NPs), they are more stable in air and have the benefits of possessing a much lower optical absorption in the visible region [33], suitable for biotechnologies. For water remediation applications, γ-Fe_2_O_3_NPs can act as photocatalysts to break down and remove various organic contaminants. To date, various nanocomposite-based γ-Fe_2_O_3_NPs have been used as adsorbents to remove different metallic- and organic-based contaminants from aqueous solutions (drinking water, groundwater, wastewater, and acid mine drainage) with significant adsorption efficiency. The pollutants include heavy metal ions and several dyes (e.g., methyl orange (MO), methylene blue (MB), rose bengal, Congo red (CR), brilliant cresyl blue, thionine, and Janus green B) [34,35]. Although several worthwhile water purification studies have been performed using magnetogels [26,36,37,38,39], only a few water remediation applications of peptide-based magnetogels are known. Thus, it is worth studying this topic to gain more insight toward the development of advanced and smart biocompatible adsorbents.

Concerning magnetogels synthesis, several methods have been introduced, such as blending [40], grafting [41], in situ precipitation [42], and swelling [43]. Among these methods, the swelling strategy is considered as an in situ methodology, and the magnetogels are prepared in one step. On the other side, blending and grafting methods are known as “ex situ” strategies, and the magnetogels are synthesized in more than one step [27]. Regarding the blending method, it is based on sequential syntheses of the components, starting with the magnetic NPs, which are then blended with hydrogel precursors to make the resultant magnetogels. Despite the simplicity of the blending method, the magnetic nanoparticles may have an interfering effect on hydrogel formation and also have a negative influence on the final structure of the gel. Moreover, lack of proper stabilization of nanoparticles can result in their heterogeneous distribution or diffusion out of the gel upon swelling [27]. Based on the interaction of MNPs with the hydrogel network, magnetogels are classified into class I and class II, as discussed by Weeber et al. [27]. In class I magnetogels, nanoparticles have a weak interaction with the hydrogel network (also called blends) through physical interactions, e.g., nanoparticles embedded in the aqueous compartments or adsorbed onto the fibers. On the other hand, class II magnetogels exhibit strong interactions among MNPs and hydrogel fibers, through covalent bonding or strong physical forces [27]. From an industrial point of view, the synthesis of novel magnetic hybrid sorbents with improved properties in terms of sorbent efficiency or ability to simultaneously introduce different extraction approaches is highly desirable.

Hence, due to the importance of magnetogels, we studied the synthesis, characterization, and potential water remediation application of novel peptide-based magnetogel nanocomposites by ex situ encapsulation of polyacrylic acid (PAA)-modified iron oxide magnetic nanoparticles (γ-Fe_2_O_3_NPs) into a peptide hydrogel matrix (γ-Fe_2_O_3_NPs@HG). The γ-Fe_2_O_3_NPs were synthesized by the well-known co-precipitation method, and for the hydrogel matrix, Fmoc-Phe and diphenylalanine (Phe_2_) were used as starting materials for the hydrogelator synthesis through an enzymatic reaction [44]. The magnetogel nanocomposite was characterized using different spectroscopic, morphological, and structural techniques and applied to the separation of Cr(III), Co(II), and Ni(II) from aqueous solutions. The effects of contact time and an external magnetic field on the adsorption efficiency of the different contaminants were investigated.

## 2. Results and Discussion

### 2.1. Preparation of γ-Fe_2_O_3_NPs and γ-Fe_2_O_3_NPs@HG Magnetogel

**Figure 1:**
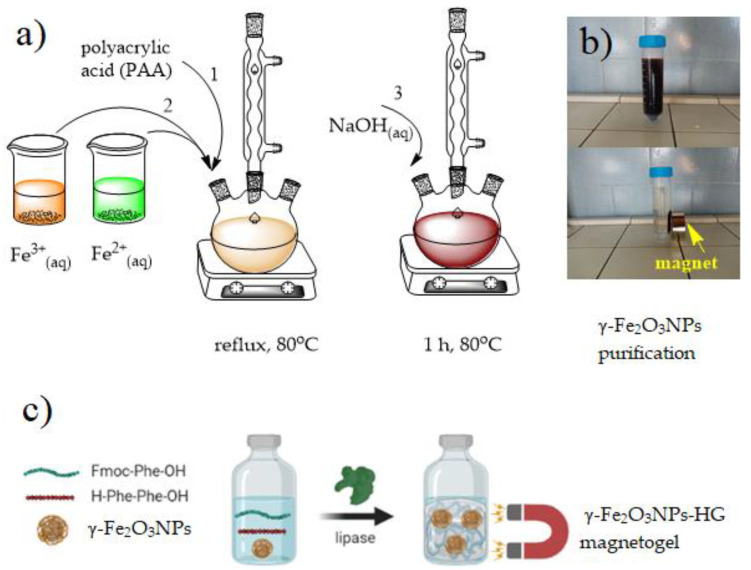
(**a**) Schematic in situ synthesis of PAA-stabilized γ-Fe_2_O_3_NPs; (**b**) separation of γ-Fe_2_O_3_NPs with an external magnetic field (the enlarged image is provided in SI as Figure S1); (**c**) illustration of the ex situ incorporation of γ-Fe_2_O_3_NPs into the peptide-based hydrogels γ-Fe_2_O_3_NPs@HG (blending method).

Co-precipitation is known as a simple and cheap method to synthesize magnetic iron oxide NPs (γ-Fe_2_O_3_NPs) from aqueous solutions of Fe(II) and Fe(III) by the addition of a base as a precipitating agent at mild temperature, and a large amount of NPs can be prepared by this method. The co-precipitation process, schematized in Figure 1a, does not require organic solvents or toxic precursor iron complexes and proceeds at temperatures below 100 °C. More importantly, it can be developed and scaled up from lab to industry due to its simplicity, reproducibility, and eco-friendly reaction conditions. However, this method sometimes suffers from a lack of control over particle size distribution, probably because of the complicated set of pathways that lead to the formation of NPs [45]. The general mechanism for the formation of MNPs first involves hydroxylation of the ferrous and ferric ions to form Fe(OH)_2_ and Fe(OH)_3_, respectively. These two low-soluble hydroxides (K_ps_ (25 °C) = 7.9 × 10^−15^ and 6.3 × 10^−38^, for ferrous and ferric hydroxide, respectively [46]) can be obtained at alkaline pHs (pH > 8), and when NaOH is used as the precipitating agent, a black colloidal solution of iron containing NPs is formed instantaneously. By applying a 2:1 molar ratio of Fe(III):Fe(II) and an oxygen-free environment, magnetite NPs (Fe_3_O_4_NPs) are the main product of this reaction through the following possible reactions (Equations (1)–(4)) [45]:

Fe(III) + 3OH^−^ → Fe(OH)_3_ (1)

Fe(OH)_3_ → FeOOH + H_2_O (2)

Fe(II) + 2OH^−^ → Fe(OH)_2_ (3)

2FeOOH + Fe(OH)_2_ → Fe_3_O_4_NPs↓+ 2H_2_ (4)

Magnetite shows an inverse (or normal) spinel crystal structure, and its unit cell contains 32 O^2−^ anions, 8 Fe(II), and 16 Fe(III) cations. Due to the presence of reduced iron (Fe(II)) in this crystal structure, Fe_3_O_4_NPs are easily subject to oxidation and are transformed to a more stable maghemite phase (γ-Fe_2_O_3_NPs) by the following equation (Equation (5)) [47]:

3Fe_3_O_4_NPs + 0.5O_2_ + 2H^+^ → 4γ-Fe_2_O_3_NPs + Fe^2+^ + H_2_O (5)

Another important aspect of iron-based NPs is their colloidal stability after the synthesis. Due to their magnetic properties, iron-based NPs are more vulnerable to agglomeration because of the magnetic attraction among particles. In general, colloidal stability is the result of a balance between repulsive interactions (steric and electrostatic) and attractive forces (Van der Waals, dipolar, and magnetic), which can be influenced by the medium parameters, including composition, pH, and ionic strength [48]. To enhance their colloidal stability, MNPs should be stabilized by steric, electrostatic, or a combination of these repulsive forces. In the electrostatic stabilization, the repulsive forces between the NPs originate from likewise charges [49], and for the steric stabilization, the presence of large molecules provides a repulsive hindrance for the surface of NPs [22]. Steric stabilization is usually favored because it is less sensitive to medium parameters and therefore more suitable when MNPs are in contact with complex media [50,51,52]. To this aim, several small and large stabilizing agents have been applied for the surface functionalization of MNPs, such as polymers (polytrolox ester, PAA, and polyacrylic-co-maleic acid), natural antioxidants (green tea polyphenols, curcumin, quercetin, and anthocyanins) and organic or inorganic acids (gallic, ascorbic, citric, and humic acid) [53]. Among these stabilizers, the functionalization of magnetic NPs with PAA provides both steric and electrostatic effects on the NPs’ surface [54]. The electrostatic effect of PAA originates from its carboxylate groups, and the steric effect from its polymeric nature. For water purification applications, PAA is known for its ability to absorb a large amount of water and is used as a superabsorbent. Another advantage of PAA is its biocompatibility which is highly desirable [40]. For the PAA functionalization of NPs, two methods are generally used: in situ and post (ex situ) surface coating. For in situ functionalization, PAA is used simultaneously with the iron precursors during the synthesis of magnetic NPs, and both synthesis and functionalization occur simultaneously in one step. For the post (ex situ) method, PAA is added to pre-synthesized NPs in a separate step (next step) from the synthesis. Generally, the in situ method is more preferable due to the inhibition of particle growth in a high concentration of PAA. Also, the high hydrophilicity and colloidal stability induced by PAA stabilization of magnetic NPs can decrease the long gelation time of supramolecular magnetogels. For instance, it was reported that the stabilization of iron oxide NPs with polyacrylic acid allowed homogeneous encapsulation of NPs up to 30 m/m% in both Npx-L-Asp-Z-ΔPhe-OH and Npx-L-Tyr-Z-ΔPhe-OH hydrogels containing non-canonical amino acids [27]. These are some important benefits of PAA for its applications as composite adsorbents. For instance, PAA-functionalized magnetic magnetite particles have been used as an adsorbent for basic dyes [53,55,56]. For the above reasons, we synthesized the magnetic NPs stabilized by PAA using the in situ strategy to obtain small and colloidally stable γ-Fe_2_O_3_NPs, followed by preparation of γ-Fe_2_O_3_NPs@HG magnetogel.

The isolation of γ-Fe_2_O_3_NPs was first confirmed by the visual magnetic behavior of the purified precipitates. As can be seen in Figure 1b and Figure S1, the particles are strongly attracted to the external magnet and easily redispersed after removing the magnet. To confirm the stabilizing effect of PAA, uncoated magnetic NPs were also synthesized, using the same reaction conditions but without the presence of PAA. For the synthesis of γ-Fe_2_O_3_NPs@HG magnetogels, we have used the blending strategy mentioned in the introduction section as a well-known method to synthesize magnetogels [25,27]. This method is based on sequential syntheses of the components, starting with the magnetic NPs, which are then blended with hydrogel precursors to make the resulting magnetogels. The formation of the hydrogel followed a well-assessed procedure, using a microbial lipase to catalyze the synthesis in water of self-assembling peptides generated by the peptide bond formation between 9-fluorenylmethoxycarbonyl-phenylalanine (Fmoc–Phe) and the dipeptide diphenylalanine (Phe_2_) (Figure 1c) [57].

### 2.2. Raman, FT-IR/ATR, and XPS Characterization of γ-Fe_2_O_3_NPs

**Figure 2:**
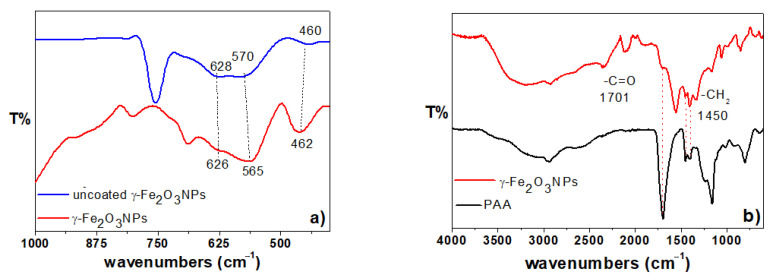
(**a**) FT-IR spectra of uncoated γ-Fe_2_O_3_NPs NPs (blue line) and PAA-stabilized γ-Fe_2_O_3_NPs (red line); (**b**) FT-ATR of PAA-stabilized γ-Fe_2_O_3_NPs (red line) and PAA (black line).

Fourier transform-infrared (FT-IR) and Raman spectroscopies are two commonly used techniques to characterize iron oxide nanoparticles, as they can provide information on the oxide phase through detection of phonon modes [58]. The FT-IR spectra of bare and PAA-stabilized γ-Fe_2_O_3_NPs are presented in Figure 2a in the 1000–400 cm^−1^ region, containing information about the phase of both NPs. Based on literature, the magnetite phase has a sharp and symmetrical vibration at around 571 cm^−1^, assigned to the Fe–O bonds present in tetrahedral and octahedral sites of the spinel structure. It is a general characteristic band of iron oxide NPs, and its sharpness clarifies the pure defect-free phase. In maghemite, this band splits into two characteristic vibrations, due to the creation of vacancy defects and vanishing of Fe(II) ion from the octahedral sites upon the formation of γ-Fe_2_O_3_, causing a decrease in Fe–O bond length, and hence, corresponding splitting of the band occurs [59]. It should be mentioned that both phases (magnetite or maghemite) show a weak band in the 440–460 cm^−1^ region [58]. Regarding the crystal structure, also group theory theoretically predicts that if the γ-Fe_2_O_3_NPs have a spinel crystal, then there are four T_1_ modes expected at 212, 362, 440, and 553 cm^−1^ [60], of which we were able to only detect the highest two frequencies with our experimental setup. Both spectra of γ-Fe_2_O_3_NPs clearly show the two T_1_ modes around 460 and 570 cm^−1^ for uncoated γ-Fe_2_O_3_NPs, and 462 and 565 cm^−1^ for the PAA stabilized γ-Fe_2_O_3_NPs, confirming a spinel crystal structure for both NPs of our experiment. Hence, in both spectra, the formation of γ-Fe_2_O_3_NPs was confirmed from the broadening and splitting of the band into 628 and 570 cm^−1^ for uncoated γ-Fe_2_O_3_NPs, and 626 and 565 cm^−1^ for PAA-stabilized γ-Fe_2_O_3_NPs. Regarding the PAA coating and its interaction with the surface of NPs, the FT-ATR data are shown in Figure 2b for PAA-stabilized γ-Fe_2_O_3_NPs (red line) and pristine PAA (black line) in the 4000–600 cm^−1^ region. The FT-ATR spectrum of PAA stabilized γ-Fe_2_O_3_NPs is not a simple superposition of the PAA spectrum, and the relative intensities of the main vibrational bands show some changes, which suggests that PAA alters its symmetry when it attaches to the NPs. The main features of these spectra are summarized in Table 2, which shows the characteristic frequencies of free PAA, including the carbonyl stretching (−C=O) at 1669 cm^−1^, −CH_2_ scissoring at 1446 cm^−1^, and the −C−O stretching at 1236 cm^−1^, as well as the symmetric stretching frequencies of the carboxylate ions (−COO^−^) at 1402 cm^−1^. Compared to the PAA spectrum, the PAA-stabilized γ-Fe_2_O_3_NPs spectrum shows all these characteristic bands (except –C–O stretching) with a maximum of 6 cm^−1^ shift. There is also an additional band for the PAA-stabilized γ-Fe_2_O_3_NPs at around 1556 cm^−1^, assigned to –COO^–^ asymmetric stretching. These results confirm the surface functionalization of NPs with PAA, and based on the small shifts observed in frequencies of PAA-stabilized γ-Fe_2_O_3_NPs (compared to the PAA spectrum), a physical interaction can be proposed between the negatively charged PAA (–COO^–^) and positive surface Fe(III) ions of NPs [61].

As mentioned above, Raman spectroscopy can discriminate iron oxide phases because they exhibit distinct Raman signatures originating from their different oxidation states [62]. Hence, Raman spectroscopy was used as a complementary technique to better understand the structure of the synthesized γ-Fe_2_O_3_NPs.

**Figure 3:**
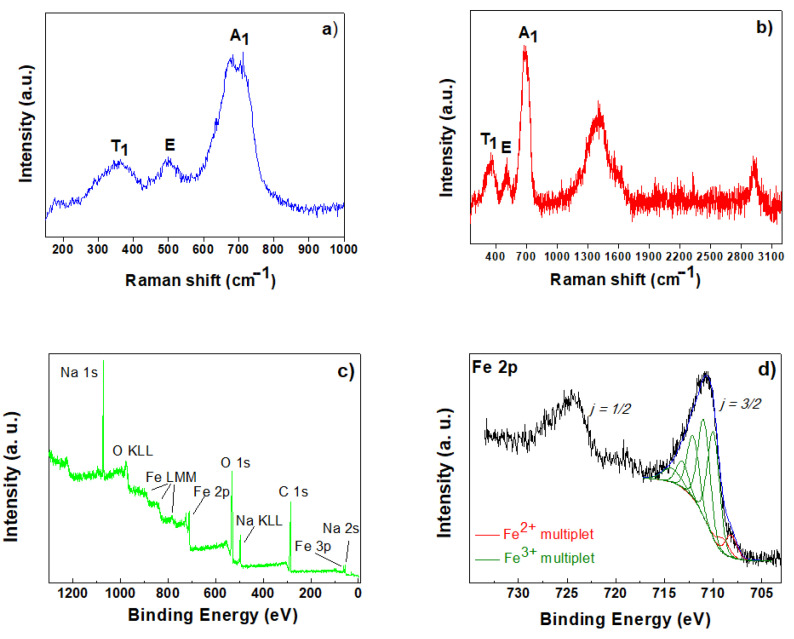
(**a**) Raman spectra of uncoated γ-Fe_2_O_3_NPs; and (**b**) PAA-coated γ-Fe_2_O_3_NPs; (**c**) XPS survey spectrum of PAA-coated γ-Fe_2_O_3_NPs; (**d**) XPS Fe2p spectrum of PAA-coated γ-Fe_2_O_3_NPs.

The Raman spectra of the synthesized uncoated γ-Fe_2_O_3_NPs and PAA-coated γ-Fe_2_O_3_NPs are shown in Figure 3a,b, respectively. The results clearly match Raman spectra for maghemite previously reported in the literature [58,63], with the three broad observed Raman active phonon modes at around 350 cm^−1^ (T_1_), 500 cm^−1^ (E), and 700 cm^−1^ (A_1_). Regarding the PAA-coated sample, we clearly see the Raman modes at higher wavenumbers, with two main bands centered at around 1400 and 2927 cm^−1^, assigned to –COO^−^ (symmetric) and –CH/–CH_2_ stretching bands, respectively [64]. These results are in accordance with the FT-IR spectra and confirm the presence of maghemite phase for the PAA-coated γ-Fe_2_O_3_NPs [58,62,65,66,67,68].

The maghemite phase is also confirmed by XPS results (Figure 3c,d) [62,69,70,71,72,73], and the PAA-coated γ-Fe_2_O_3_NPs sample was analyzed with XPS in order to ascertain the chemical composition of the inorganic core structure. Figure 3c shows the XPS spectrum, where the signals due to ionization of Fe, C, O, and Na are visible, the latter resulting from the use of NaOH in the synthesis of PAA-coated γ-Fe_2_O_3_NPs. Figure 3d shows the Fe 2p spectrum of PAA-coated γ-Fe_2_O_3_NPs, which is composed of two rather broad spin-orbit split components (j = 3/2 and 1/2), whose maxima are separated by a ΔE_so_ ~ 13.6 eV. This spectrum was curve-fitted in order to determine the oxidation state of the iron species. The curve-fitting procedure was conducted following the work by Grosvenor et al. [69,70], who applied a Shirley background removal to the 2p_3/2_ envelope and successfully used the Gupta and Sen (GS) multiplets calculated for free metal ions [71,72] to account for electrostatic interactions in high-spin Fe(II) and Fe(III) compounds [69,70]. Also in the present case, a five-fold GS multiplet for Fe(III) compounds was used in the curve-fitting (green curves in Figure 3d, first component at 710.05 eV binding energy), achieving a good match with the experimental data. This agreement strongly supports the attribution of this signal to γ-Fe_2_O_3_, as reported by Grosvenor et al. A further two-fold multiplet was added at low binding energy (red curves in Figure 3d, first component at 708.17 eV binding energy), in order to better reproduce the experimental signal. This multiplet appears also in the spectrum by Grosvenor et al. as a “pre-peak” and might stem from residual Fe(II) high-spin components possibly present in γ-Fe_2_O_3_ [69]_._

### 2.3. DSL and UV-Vis Characterization of γ-Fe_2_O_3_NPs

**Figure 4:**
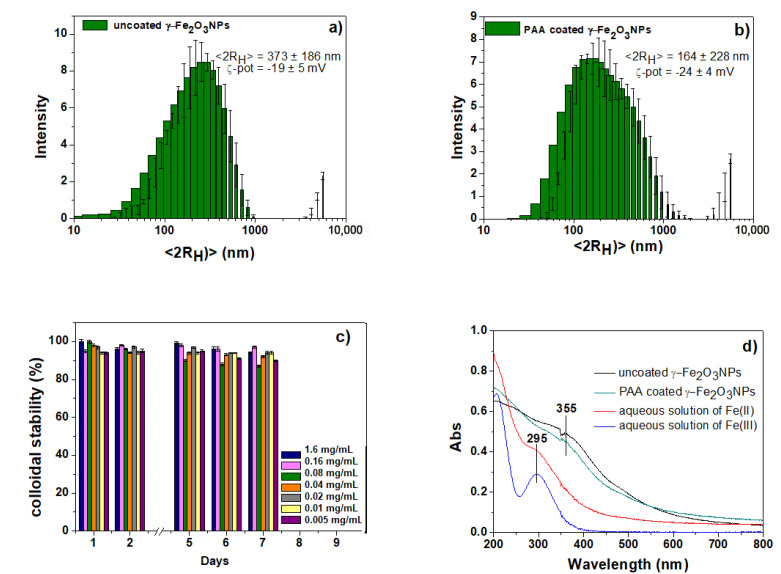
(**a**,**b**) DLS results of the two bare and PAA-coated γ-Fe_2_O_3_NPs; (**c**) stability tests of PAA-coated γ-Fe_2_O_3_NPs assessed by following the maximum absorption peak at 355 nm; (**d**) UV-Vis spectra of freshly prepared precursors ions (Fe^2+^_(aq)_ and Fe^3+^_(aq)_) and the two NPs.

The hydrodynamic size and surface charges of the two γ-Fe_2_O_3_NPs systems (bare and coated with PAA) were studied by DLS and ζ-potential techniques (Figure 4a,b). Compared to uncoated γ-Fe_2_O_3_NPs, PAA-coated γ-Fe_2_O_3_NPs show smaller size and higher negative surface charge, which can be a good indication of PAA coating and, more importantly, PAA’s stabilizing effect on the NPs. Using PAA in the in situ synthesis of NPs allows for controlling the size of γ-Fe_2_O_3_NPs because the attached PAA moiety provides steric (by its polymeric nature) and electrostatic (by its –COO^−^ group) stabilizations, thus permitting γ-Fe_2_O_3_NPs dispersion in aqueous media [54]. In fact, the presence of PAA on the surface of γ-Fe_2_O_3_NPs prolongs the colloidal stability by slowing down the agglomeration process. Another major advantage of PAA is that the carboxylic acid-enriched surfaces of PAA-coated γ-Fe_2_O_3_NPs may provide a platform for attaching these NPs to other systems to prepare multifunctional nanohybrids. UV-Vis spectra of PAA-coated γ-Fe_2_O_3_NPs (1.6–0.005 mg/mL) were monitored over a 7-day period to characterize their colloidal stability against agglomeration and sedimentation [74,75,76]. As can be seen in Figure 4c, results show no significant changes in the intensities, indicating the stability of the preparations against aggregation. The images of three PAA-coated γ-Fe_2_O_3_NPs suspensions (1.6, 0.16, and 0.08 mg/mL) are shown in Figure S2a, in which no precipitation was observed, consistent with the UV-Vis spectra (Figure 4c). Also, the colloidal stability of higher concentrations of PAA-coated γ-Fe_2_O_3_NPs (10 mg/mL) was visually monitored over one week, and a remarkable stability of the NPs against sedimentation was observed. Conversely, uncoated γ-Fe_2_O_3_NPs showed a fast aggregation and sedimentation that were visually detected, even at low concentrations (Figure S2c). These results are consistent with previous studies on the use of organic molecules as stabilizing agents for MNPs. However, the uncoated NPs showed a limited amount of precipitation in the vials (Figure S2c), demonstrating the positive long-term stabilizing effect of PAA-coated γ-Fe_2_O_3_NPs (Figure 4c). These results are comparable and consistent with the literature [27,40,53,54,55,56] about the stabilizing effect of PAA on γ-Fe_2_O_3_NPs, explained in Section 2.1.

The UV-Vis spectra of both NPs systems show the presence of a new absorption band at around 355 nm (Figure 4d), which is different compared to the spectra of precursors ions (Fe^2+^_(aq)_ and Fe^3+^_(aq)_) and assigned to the band gap of maghemite derived from O(2p) → Fe(3d) transitions [77,78]. In the freshly prepared samples of both NPs, shoulder peaks centered at around 450 nm are observed due to the presence of a minor amount of Fe_3_O_4_NPs, which then oxidized to the maghemite phase, and are not seen in the results of previous section (Section 2.2).

The peaks of Fe^3+^_(aq)_ and Fe^2+^_(aq)_ are assigned to charge-transfer electronic transition in octahedral aquo complexes [79,80,81]. Regarding these UV-Vis spectra of the Fe^3+^_(aq)_/Fe^2+^_(aq)_, it is worthy to explain that their electronic transition is largely governed by their d-electron configuration. The charge-transfer transitions are both spin- and Laporte-allowed and occur in the UV region, which is closely related to the strength of the applied ligand field (10D*q*) [82].

### 2.4. FESEM-EDS Characterization of γ-Fe_2_O_3_NPs and γ-Fe_2_O_3_NPs@HG Magnetogel

**Figure 5:**
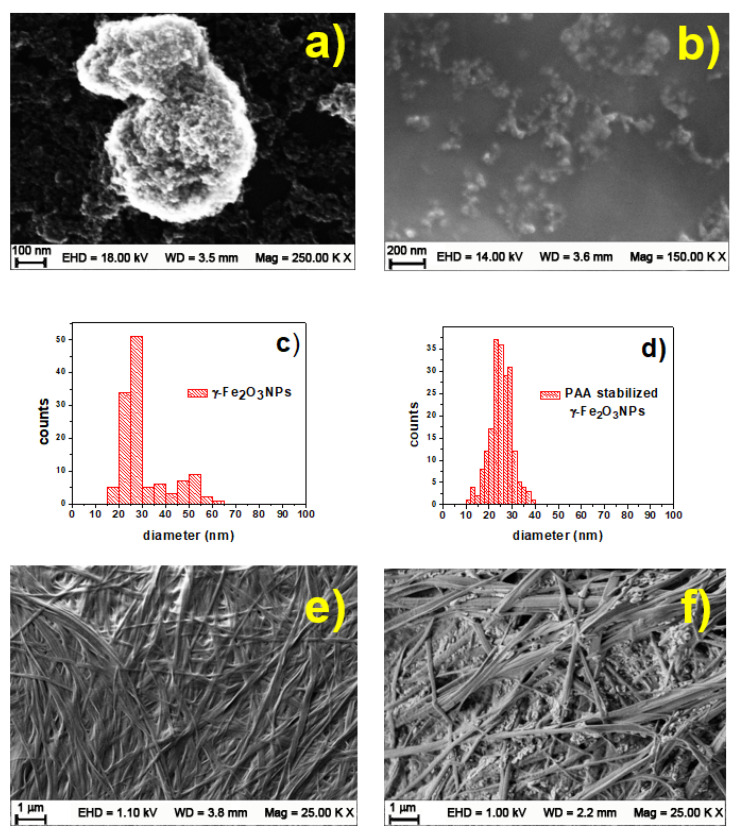
(**a**) FESEM results of uncoated γ-Fe_2_O_3_NPs and (**b**) PAA-coated γ-Fe_2_O_3_NPs; (**c**) size histogram of γ-Fe_2_O_3_NPs and (**d**) PAA-coated γ-Fe_2_O_3_NPs; (**e**) FESEM of peptide hydrogel alone and (**f**) γ-Fe_2_O_3_NPs@HG magnetogel. EDS data of PAA-coated γ-Fe_2_O_3_NPs are reported in the supporting section (Figure S3).

The solid-state morphology and size of the γ-Fe_2_O_3_NPs were evaluated using Field Emission Scanning Electron Microscopy (FESEM) (Figure 5a,b). The FESEM image of uncoated γ-Fe_2_O_3_NPs demonstrates a grain-like morphology with a size range of 15–65 nm (Figure 5a,c), and a little aggregation is detected in the image. In agreement with DLS results, the FESEM images of PAA-coated γ-Fe_2_O_3_NPs show smaller particles (Figure 5b,d, 10–40 nm). This result is consistent with the general mechanism of γ-Fe_2_O_3_NPs formation including a nucleation step in the beginning of co-precipitation, followed by nuclei growth and coalescence. This mechanism is supported by our results, in which both DLS and FESEM analyses indicate that the γ-Fe_2_O_3_NPs obtained without PAA have a larger average size compared to the particles in the presence of PAA. EDS elemental analyses showed the presence of C element, which is due to the presence of PAA, previously confirmed by the FT-IR/ATR and Raman results (Figure S3). For both γ-Fe_2_O_3_NPs (with and without PAA), we see smaller distribution particles, compared with the DLS results, relating to the differences between these two techniques. In fact, FESEM probes the electron-rich part of the particle in the solid state; then only the inner core can be seen, and the result obtained would be smaller. On the other hand, the DLS measures a hydrodynamic diameter based on the diffusion of the particles in the solutions, and in most cases, we see larger nanoparticles due to the hydrodynamic layer. FESEM images of the hydrogel and magnetogel are shown in Figure 5e,f, both exhibiting the typical fibrillar structure, suggesting that the presence of PAA-coated γ-Fe_2_O_3_NPs does not change the macromolecular structure of the gel. Also, the small particles seen in the magnetogel image can be assigned to γ-Fe_2_O_3_NPs.

### 2.5. Rheological Studies and Swelling Ability

**Figure 6:**
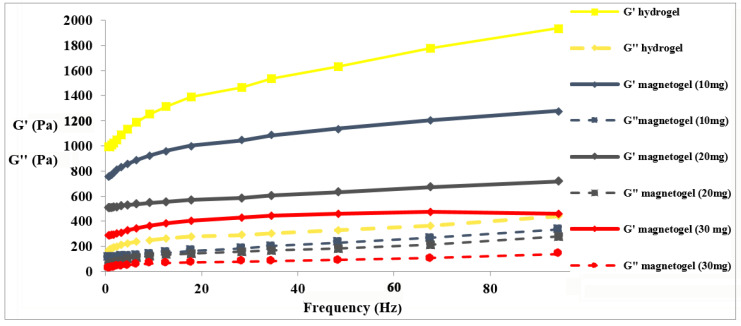
Frequency sweep of the Fmoc-Phe_3_ hydrogel (HG) and γ-Fe_2_O_3_NPs embedded in peptide magnetogel (γ-Fe_2_O_3_NPs@HG) at different concentrations.

The viscoelastic behavior and the adsorption properties of hydrogel materials are tightly correlated properties. The goal of the rheological analyses was to understand how the presence of PAA-coated γ-Fe_2_O_3_NPs and their concentration could modulate the viscoelastic behavior of the hydrogels. As can be seen in Figure 6, for all systems, the experimental curves show a typical trend of a viscoelastic gel-like material, characterized by G′ values much larger than G″ values [83]. All the magnetogels showed lower mechanical strength compared to the hydrogel alone, and a concentration-dependent trend is observed for the magnetogels’ mechanical strength, in which the highest concentration of γ-Fe_2_O_3_NPs (30 mg/mL) had the lowest storage modulus. The decrease in the strength of the magnetogels may be due to the presence of PAA-coated γ-Fe_2_O_3_NPs with different sizes, morphologies, and surface charges. The negatively charged PAA-coated γ-Fe_2_O_3_NPs could expand the hydrogel network and increase the number of pores and free spaces, lowering the mechanical strength. The PAA-coated γ-Fe_2_O_3_NPs can interact with some hydroxyl and amine groups of the hydrogel using Fe(III) and PAA moieties expanding the internal network structure [84,85,86,87,88]. The swelling abilities of hydrogel and magnetogel samples were measured and are summarized in Table 3. All magnetogels showed higher swelling behavior in comparison to the native peptide-based hydrogel. This result may be attributed to the interaction of the hydrogel networks with PAA-coated γ-Fe_2_O_3_NPs, neutralizing the repulsions in the networks and resulting in the penetration of more water in order to compensate for the buildup of osmotic ion pressure [44,89]. Another water-adsorbing moiety is the attached PAA [40]. These results are consistent with the rheological studies, and there is an inverse relationship between them, meaning that the higher mechanical strength causes the lower swelling ability of the gels [38]. For all successive removal applications, we used the 10 mg/mL PAA-coated γ-Fe_2_O_3_NPs magnetogels because of their higher mechanical strength compared to the other two magnetogels.

### 2.6. Magnetogels Application in the Removal of Metallic Cations

A simple and straightforward procedure of absorption was applied to Co(II), Ni(II), and Cr(III) aqueous solutions in the presence of fixed quantities of HG and γ-Fe_2_O_3_NPs@HG, following the UV-Vis peak arising from the metal ions over time, also comparing the effect of an external magnetic field (1.42–1.47 T). In the following paragraphs, the results of the studies are reported.

#### 2.6.1. Co(II) Removal Studies

**Figure 7:**
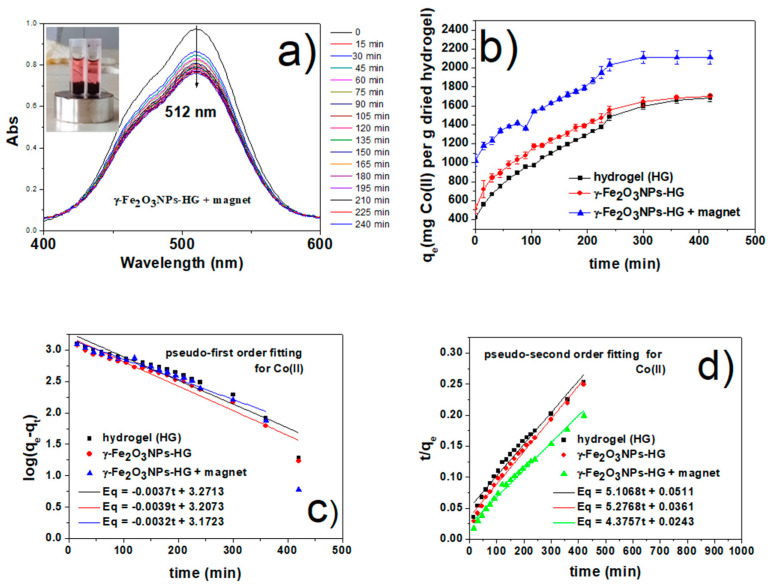
(**a**) UV-Vis study of Co(II) adsorption by γ-Fe_2_O_3_NPs@HG upon magnetic field application (the UV-vis spectra for the peptide HG and γ-Fe_2_O_3_NPs@HG are reported in the Supporting Information section, together with the calibration curve for Co(II) aqueous solutions); (**b**) adsorption capacity of HG, γ-Fe_2_O_3_NPs@HG, and γ-Fe_2_O_3_NPs@HG upon magnetic field application versus time; (**c**) fit of kinetic data to pseudo-first order model and (**d**) pseudo-second order model for Co(II).

Co(II)_(aq)_ has a broad metal-based absorption peak in the visible region centered at 512 nm [90]. Over the whole removal process, the intensity of this peak decreases without significant change in the wavelength for all the three adsorbents (Figure 7a and Figure S4) tested, i.e., HG, γ-Fe_2_O_3_NPs@HG, and γ-Fe_2_O_3_NPs@HG upon magnetic field application. In particular, in Figure 7a, the UV-Vis spectra of γ-Fe_2_O_3_NPs@HG upon magnetic field application is reported. The adsorption capacity of these systems was monitored as a function of contact time (Co(II)_initial_ = 61 mg/mL), and q_t_ (in mg g^−1^) plots are shown in Figure 7b, with contact times ranging from 0 to 480 min. The adsorption capacities follow the trend γ-Fe_2_O_3_NPs@HG upon magnetic field application > γ-Fe_2_O_3_NPs@HG > HG, confirming the enhancing effect of γ-Fe_2_O_3_NPs on the adsorption. The interaction of magnetogel with an external magnet further increases the capacity, compared to that of the magnetogel. For each adsorbent, a fast Co(II) removal was observed in the first 15 min, which is due to the large number of active sites available on the hydrogel [91]. This rapid adsorption might be due to chemical rather than physical adsorption because of the possible complexation between the suitable functional groups of hydrogels and Co(II) ions. This phenomenon was already reported in several studies of Co(II) adsorption [16,92,93]. Then, their adsorption gradually slowed until reaching equilibrium after 480 min for all systems. At equilibrium, the capacity of γ-Fe_2_O_3_NPs@HG upon magnetic field application is about 1.25 times higher than that of the other two adsorbents. Total adsorption efficiencies were estimated, and the results show that γ-Fe_2_O_3_NPs@HG and γ-Fe_2_O_3_NPs@HG upon magnetic field application increase the removal efficiency by 0.3% and 5.3%, respectively (Table 4), in comparison with hydrogel alone. Under these experimental conditions, the external magnetic field has a relevant effect on the adsorption capacity and efficiency of the hydrogel.

As time passes, the removal speeds decrease, owing to more active sites occupied by Co(II) ions. To better understand the adsorption kinetics, two well-known kinetic models, pseudo-first order and pseudo-second order [91], were applied and evaluated using the given linearized equations (Equations (9) and (10)), as provided in Section 4.8. In general, the pseudo-first order model describes a reversible adsorption between solid and liquid phases [94], claiming physisorption rather than chemisorption. Conversely, the second-order model mainly suggests a chemical adsorption of adsorbates onto the adsorbents [95], in which chemical bonding occurs among the metal ions and polar functional groups of the adsorbents. Two kinetic plots are shown in Figure 7c,d, and the subsequently calculated kinetics parameters are summarized in Table 5. Considering the correlation coefficient, R^2^ better-fitted straight lines were obtained from the pseudo-second order relation for all the adsorbents, compared to those obtained for the pseudo-first order plots. Moreover, the calculated equilibrium adsorption capacities, q_e_, obtained from the pseudo-second order equation were much closer to the experimental trend seen in Figure 7b. The results suggest that the adsorption of Co(II) onto our synthesized HG and γ-Fe_2_O_3_NPs@HG systems occurs through chemisorption, in agreement with previous works [96]. Also, we can clearly see the significant effect of magnetic interaction on the speed of adsorption, as shown in Table 5.

The chemisorption of Co(II) can be interpreted based on the coordination chemistry of Co(II) aquo complexes, which are kinetically labile species. For substitution reactions, the transition metal kinetics are governed by their electronic configurations. For octahedral complexes, the d orbitals split into high energy e_g_ (d_z_^2^ and d_x_^2^_−y_^2^) and low energy T_2g_ (d_xz_, d_yz_, d_xy_) levels, of which e_g_ orbitals show anti-bonding characters. Due to the high-spin d^7^ configuration of Co(II) aquo complexes having the T_2g_^5^ e_g_^2^, they also exhibit the Jahn-Teller effect (z-out), which is a geometric distortion of a non-linear molecular system that reduces its symmetry and energy, seen in the complexes having the occupied e_g_ levels. Therefore, the Co(II) ions in the dissolution process have high lability and are vulnerable to the chemical substitution reaction to achieve their stable electronic configurations. In fact, the presence of Fmoc-Phe and diphenylalanine (Phe_2_) in the hydrogel network can provide the chelating moiety (through their nitrogen and oxygen atoms) for the Co(II) ions, resulting in stable Co(II) complexes [97].

Regarding the enhancing effect of γ-Fe_2_O_3_NPs nanoparticles, it is consistent with previous studies on magnetogels [98,99] and is related to the fact that NPs embedded in hydrogel can increase the cross-linking degree and porosity of the gel, providing a channel for the entry, exit, and adsorption of some substances [28]. More importantly, the interaction of γ-Fe_2_O_3_NPs@HG with the external magnet further enhances the adsorption. As is well-known, magnetogels can exhibit an on/off effect on the hydrogel pores [27]. In fact, swelling or shrinking states of γ-Fe_2_O_3_NPs@HG can be influenced by the magnetic dipole-dipole orientation of γ-Fe_2_O_3_NPs toward the external magnetic field [100] and can increase the permeability of Co(II) into the hydrogel network for the chemisorption [89,101].

#### 2.6.2. Ni(II) Removal Studies

**Figure 8:**
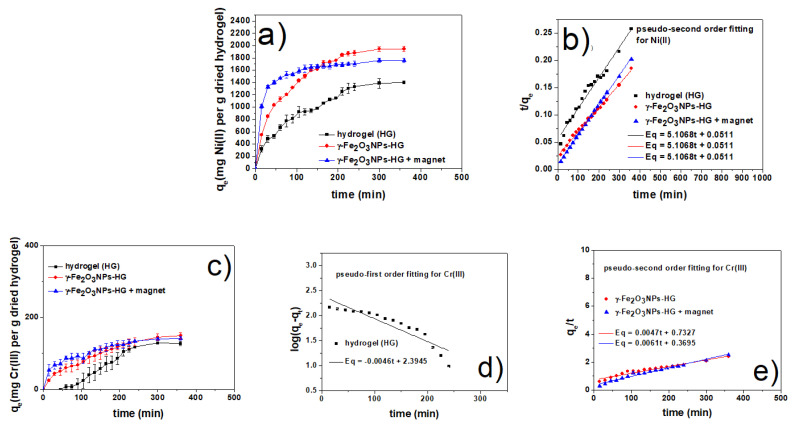
(**a**) Ni(II) adsorption capacity of HG, γ-Fe_2_O_3_NPs@HG, and γ-Fe_2_O_3_NPs@HG + magnet versus time; (**b**) fit of kinetic data to the pseudo-second-order model for Ni(II); (**c**) Cr(III) adsorption capacity of HG, γ-Fe_2_O_3_NPs@HG, and γ-Fe_2_O_3_NPs@HG + magnet versus time; (**d**) fit of kinetic data to the pseudo-first and (**e**) second-order models for Cr(III). (The complete UV-Vis study of Ni(II) and Cr(III) adsorption are reported in the supporting information section together with the Ni(II) and Cr(III) calibration curves.)

Ni(II)_(aq)_ complexes display a typical octahedral structure with six water ligands in the first coordination shell [102] and an absorption maximum at 394 nm due to spin-allowed transitions. Upon interaction, a decrease in the absorption band was observed without significant changes in the wavelength (see supporting information, Figure S5). The γ-Fe_2_O_3_NPs@HG dramatically enhances the adsorption of Ni(II), with and without an external magnet. All the three systems show very fast adsorptions in the early 30 min, and applying an external magnet results in reaching the equilibrium earlier, after around 150 min for γ-Fe_2_O_3_NPs@HG + magnet. Considering the effect of an external magnet on γ-Fe_2_O_3_NPs@HG adsorption ability, a plateau is reached after 150 min. Without applying the external magnet, the equilibrium is reached later (after 360 min) but with 10% higher adsorption capacity (Figure 8a). The adsorption efficiencies are summarized in Table 6, showing that the γ-Fe_2_O_3_NPs@HG and γ-Fe_2_O_3_NPs@HG + magnet systems can increase the removal efficiency by 7.3% and 5.1%, respectively, compared to the native hydrogel.

For all the three adsorbents, the speed of adsorption is higher in the beginning and slows down with time. Also, the adsorption speed of magnetogels (with and without an external magnet) is higher than that of the native hydrogel. Applying the kinetics models for the Ni(II) removal, it can be seen (Figure 8b) that the pseudo-second order model was more consistent for all these three systems (all second-order coefficients R^2^ are higher than 0.97), supporting a chemisorption mechanism for Ni(II) (Table 7).

#### 2.6.3. Cr(III) Removal Studies

Cr(III) removal showed a different pattern, compared to Co(II) and Ni(II) adsorption. It is known that the aqueous Cr(III)_(aq)_ can show three absorption peaks due to both the d→d electronic transitions [103]. In the present study, the absorption peak of 420 nm was monitored and assigned to spin-allowed transition. The UV-Vis spectra (reported in the supporting information, Figure S6) show the decrease in the 420 nm absorption band due to the removal of Cr(III). As can be seen in Figure 8c, the adsorption rate of both magnetogels γ-Fe_2_O_3_NPs@HG with and without the applied magnet is higher than that of the hydrogel HG in the first half of the experiments. However, at equilibrium, all adsorbents reach almost similar values of removal capacity (Figure 8c) (120–130 mg/g). Similar to the Co(II) and Ni(II) results, here also we clearly see the higher speed of magnetogels in removing the contaminant, further enhanced by the presence of a magnetic field. Table 8 reports the removal efficiencies for all the systems.

Kinetic studies provided us further detailed information on Cr(III) removal. The fitting results of the kinetic models are given in Table 9. For the hydrogel, the correlation coefficient (R^2^) provided by the pseudo-first order model is much higher than that of the pseudo-second order, suggesting a physical mechanism for the adsorption of Cr(III) (Figure 8d). This can be explained by the electronic configuration of Cr(III) aqua complexes (d^3^), which are kinetically inert and have a low rate for the chemical substitution reaction in normal conditions. The physical mechanism might be due to the electrostatic interaction of positively charged Cr(III) ions with the negatively charged hydrogel network [24,44]. Conversely, the data obtained with both magnetogels (with and without magnet) fit with a pseudo-second order model, suggesting that the presence of γ-Fe_2_O_3_NPs@HG and magnetic field can both change the hydrogel network and porosity, providing chelating conditions inside the hydrogel suitable for the formation of the chemical bonds among hydrogel hyteroatoms and Cr(III) ions [104].

## 3. Conclusions

The goal of this work was to direct attention to emerging and novel research involving magnetogel nanohybrid materials that might be relevant in future applications for the treatment of wastewater, as well as in other fields.

Generally, composite hydrogels are promising adsorbents with tunable features, and we demonstrated that the addition of effective functional groups in nanohybrid materials through chemical conjugation is a promising strategy to further improve the adsorption abilities of hydrogels. In fact, the results achieved pointed out that the presence of γ-Fe_2_O_3_NPs provides magnetic properties to the resulting nanohybrids, which can be applied for magnetic-based removal applications of contaminants, such as heavy metal ions, from aqueous phases. The results of the removal studies demonstrate that the presence of γ-Fe_2_O_3_NPs in combination with the application of an external magnetic field increases the adsorption efficiency of the hydrogel matrix for all the metal ions tested in this study; in particular, the γ-Fe_2_O_3_NPs@HG + magnet was effective to absorb up to 2111 ± 72 mg/g for Co(II), 1758 ± 39 mg/g for Ni(II), and 142 ± 5 mg/g for Cr(III).

The kinetic models showed the chemisorption of these cations onto the γ-Fe_2_O_3_NPs@HG (with and without the magnetic field). Regarding the native HG, Co(II) and Ni(II) showed chemisorption, but for the Cr(III), the results were fitted with a physical adsorption mechanism. This work showed that the peptide-based magnetogels can be introduced as promising adsorbing materials for wastewater treatment to remove heavy metals from aqueous solutions. In the future, this study could be expanded to test the recovering ability of the three adsorbing systems for the recycling of metal ions, and extensive efforts should be directed to scale up the applications and test the developed materials in practical scenarios.

## 4. Materials and Methods

### 4.1. Materials

L-Phenylalanyl-L-phenylalanine (H-Phe-Phe-OH, 98%, 312.36 g/mol) and *N*-(9-Fluorenylmethoxycarbonyl)-L-phenylalanine (Fmoc-L-phenylalanine: Fmoc-Phe-OH, 99%, 387.44 g/mol) were purchased from Bachem GmbH (Weil am Rhein, Germany) and used as received. FeCl_2_·4H_2_O (198.75 g/mol) and FeCl_3_ (162.20 g/mol) were purchased from Fluka. NaOH (39.99 g/mol) and CrCl_3_·6H_2_O (158.36 g/mol) were purchased from Carlo Erba Reagents (Cornaredo (MI), Italy). NiCl_2_·6H_2_O (129.59 g/mol) and CoCl_2_·6H_2_O (129.83 g/mol) were obtained from Alfa Aesar. Lipase from *Pseudomonas fluorescens* (PFL ≥ 20,000 U/mg) was purchased from Sigma-Aldrich (Milan, Italy) and used as received. Ultra-pure water (H_2_O_up_) was obtained using a Zeneer Power I Scholar-UV (Full Tech Instruments, Rome, Italy) apparatus. In this study, the external magnetic field was provided by a commercial neodymium-based magnet possessing 39.270 cm^3^ volume, magnetization quality of N5, and 1.42–1.47 T of magnetic strength.

### 4.2. Synthesis of γ-Fe_2_O_3_NPs@HG Magnetogels

γ-Fe_2_O_3_NPs were synthesized via the co-precipitation method in which 40 mg of FeCl_3_ and 25 mg of FeCl_2_·4H_2_O were dissolved in 25 mL of an aqueous solution of polyacrylic acid (PAA) and degassed with Ar_(g)_ for 15 min, followed by increasing the temperature to 80 °C [87]. Then, pH of this solution was increased to 11 by a fast addition of NaOH (10 M). The mixture was stirred at 80 °C for 1 h (with constant monitoring of the pH) and then cooled down to room temperature. The dark-brown colloidal solution of MNPs was collected by a strong magnet (1.42–1.47 T) and washed three times with a total volume of 150 mL of ultra-pure water to remove the excess amount of NaOH and other non-magnetic species. The resultant NPs were freeze-dried and stored at room temperature. Regarding PAA, it was synthesized by radical polymerization of acrylic acid (2 mL) in the presence of the initiator potassium persulfate (50 mg) in a total volume of 25 mL of water at 80 °C for 5 h. FmocPhe_3_ hydrogel synthesis was performed according to our previous work [44]. In brief, Phe_2_ and FmocPhe peptides were added in equimolar amounts to a colloidal solution containing 2 mL aqueous solution of the as-synthesized MNPs-PAA (10–30 mg/mL) and 420 µL of 0.5 M NaOH, stirring magnetically for 10 min. Then pH was adjusted to 7 by adding 1.5 mL of 0.1 M HCl, followed by the addition of 100 µL lipase aqueous solution (50 mg/mL), leaving the resultant mixture at 30 °C for 30 min.

### 4.3. UV-Vis, FT-IR/ATR and Raman Spectroscopies and Dynamic Light Scattering (DLS)

All UV-Vis spectra of the MNPs and Fe^2+^/Fe^3+^ ions were recorded in 1.00 cm optical path quartz cells using a Cary 100 Varian spectrophotometer. For the removal studies, we used the plastic PMMA cells. FTIR-ATR data were collected with a Bruker Vertex 70 instrument (Bruker Optics, Ettlingen, Germany) using KRS-5 cells in the 4000−400 cm^−1^ range or in ATR mode on a diamond crystal in the 4000−600 cm^−1^ spectral region. Dynamic light scattering (DLS) measurements were performed using a Malvern Zetasizer with a minimum of 10 replicates. All measurements were carried out at least three times with reporting the average value ± standard deviation. Raman spectra were run at room temperature in backscattering geometry with an inVia Renishaw micro-Raman spectrometer equipped with an air-cooled CCD detector and super-Notch filters. An Ar^+^ ion laser (λ_laser_ = 514 nm) was used, coupled to a Leica DLML microscope with a 20× objective. The resolution was 2 cm^−1^, and spectra were calibrated using the 520.5 cm^−1^ line of a silicon wafer [105].

### 4.4. X-ray Photoelectron Spectroscopy (XPS)

The XPS analysis was conducted using a modified Omicron NanoTechnology MXPS system.(Scienta Omicron GmbH, Taunusstein, Germany) Samples were excited by achromatic AlK*α* photons (h*ν* = 1486.6 eV), operating the anode at 14–15 kV and 10–20 mA. The take-off angle and pass energy were fixed at 21° and 20 eV, respectively. Samples were prepared by casting onto a hydrogenated Si(100) wafer a 20 μL drop of MNPs-PAA, and the obtained Si-supported sample was left to dry overnight and then mounted on a stainless steel sample holder for measurement [106].

### 4.5. Electron Microscopy Studies

FESEM images were performed using an Auriga Zeiss field emission scanning electron microscope supported by an energy dispersive X-ray spectroscopy detector (FESEM-EDS) instrument (Zeiss, Oberkochen, Germany). Samples were deposited onto conducting silicon stubs without the need of a conductive coating and analyzed at an accelerating voltage that avoided radiation damage.

### 4.6. Rheology Measurements

The rheological properties of three magnetogels containing different concentration of NPs and of the native hydrogel were studied by using an Anton Paar MCR 302 rotational rheometer in frequency sweep experiments, (Anton Paar, Turin, Italy) as reported previously [44].

### 4.7. Swelling Test

Swelling tests of the hydrogel samples were conducted as reported previously [44]. The swelling degree (q) was calculated by using the following equation:

q = (W_s_ − W_d_)/W_d_ (6)

where W_s_ is the weight of the hydrogel after removing the swelling solution and W_d_ is the weight of the freeze-dried sample.

### 4.8. Adsorption Experiments

Magnetogel samples were prepared in cuvettes, and 2 mL of the target solutions (Co(II), Ni(II), and Cr(III)) were cast on top of them, using different concentrations of the cations (see Figure S7). We only used the 10 mg type γ-Fe_2_O_3_NPs@HG magnetogel because of its higher mechanical strength, evidenced by the rheological characterization.

The removal studies were performed using UV-Vis spectroscopy, and the results are presented in Figure 7, Figure 8 and Figures S2–S4. For all the tested pollutants, we used three main hydrogel-based absorbents including (1) HG, (2) γ-Fe_2_O_3_NPs@HG magnetogel, and (3) γ-Fe_2_O_3_NPs@HG + magnet). We also studied the removal efficiency of γ-Fe_2_O_3_NPs alone (10 mg/mL), and no significant change was observed in the UV-Vis of solutions. The UV-Vis absorbances of the solutions were monitored over time with 15 min intervals, and the removal efficiency (RE) was estimated by absorption spectra using Equation (7) as follows:

RE (%) = (C_0_ − C_f_)/C_0_ × 100 (7)

where C_0_ is the initial concentration of pollutant and C_f_ is the concentration of pollutant in the eluted solution. The calibration curves were obtained and used for the calculations. Also, the adsorption capacities were estimated using the stock solutions of the pollutants (Co(II), Ni(II), and Cr(III)) prepared at pH 7, and their concentrations remaining in solutions after specific time intervals were determined by UV-Vis spectrophotometry. The adsorption capacity (q_e_, mg g^−1^) of the adsorbents was calculated using Equation (8) [107,108,109]:

q_e_ = (C_0_ − C_e_)/m × V (8)

where m (g) is the dried hydrogel mass, C_0_ and C_e_ (mg L^−1^) are the initial and equilibrium pollutant concentrations, and V (L) is the solution volume, respectively.

Kinetic behavior was studied using non-linear pseudo-first order and pseudo-second order kinetic models (Equations (9) and (10)) [108,110].

log(q_e_ − q_t_) = logq_e_ − *k*_1_t/2.303 (9)

t/q_t_ = 1/*k*^2^q_e_^2^ + t/q_e_ (10)

## Figures and Tables

**Table 1:** Heavy metals commonly found in industrial wastewaters, along with their health side effects and the permitted quantity in drinking water based on the World Health Organization (WHO) recommendations. Adapted with permission from Ref. [4]. Copyright 2021, Springer Nature.

Heavy Metal	Sources	Main Organ and System Affected	Permitted Amounts (μg)
Lead (Pb)	Lead-based batteries, solder, alloys, cable sheathing pigments, rust inhibitors, ammunition, glazes, plastic stabilizers	Bones, liver, kidneys, brain, lungs, spleen, immunological system, hematological system, cardiovascular system, reproductive system	10
Arsenic (As)	Electronics and glass production	Skin, lungs, brain, kidneys, metabolic system, cardiovascular system, immunological system, endocrine system	10
Copper (Cu)	Corroded plumbing systems, electronic and cables industry	Liver, brain, kidneys, cornea, gastrointestinal system, lungs, immunological system, hematological system	2000
Zinc (Zn)	Brass coating, rubber products, some cosmetics and aerosol deodorants	Stomach cramps, skin irritations, vomiting, nausea, anemia, convulsions	3000
Chromium (Cr)	Steel and pulp mills, tanneries	Skin, lungs, kidneys, liver, brain, pancreas, tastes, gastrointestinal system, reproductive system	50
Cadmium (Cd)	Batteries, paints, steel industry, plastic industries, metal refineries, corroded galvanized pipes	Bones, liver, kidneys, lungs, testes, brain, immunological system, cardiovascular system	3
Mercury (Hg)	Electrolytic production of caustic soda and chlorine, electrical appliances, runoff from landfills and agriculture, industrial and control instruments, laboratory apparatus, refineries	Brain, lungs, kidneys, liver, immunological system, cardiovascular system, endocrine and reproductive system	6
Nickel (Ni)	Nickel alloy production, stainless steel	Skin, gastrointestinal distress, lung, pulmonary fibrosis, kidney	70
^1^ Cobalt (Co)	Cement industries, polishing disc used in diamond polishing, mobile batteries, televisions (TVs), liquid crystal display TVs, computer monitors	High concentrations cause vomiting, nausea, vision problems, thyroid gland damage	N/A

^1^ The data for cobalt (Co) were taken from references [5,6].

**Table 2:** Comparison of FT-ATR peak assignments (in cm^−1^) for polyacrylic acid (PAA) in solid form and after attachment onto the γ-Fe_2_O_3_NPs surface.

PAA	PAA-Stabilized γ-Fe_2_O_3_NPs	Peak Assignment
1699	1705	–C=O (free COOH)
-	1556	–COO^−^ (asymmetric)
1446	1444	–CH_2_ scissor
1402	1408	–COO^−^ (symmetric)
1236	-	–C–O

**Table 3:** Swelling abilities of the hydrogel systems.

Samples	Swelling Degree (q)
HG	62.18 ± 0.35
γ-Fe_2_O_3_NPs@HG (10 mg/mL)	73.82 ± 0.99
γ-Fe_2_O_3_NPs@HG (20 mg/mL)	81.28 ± 0.22
γ-Fe_2_O_3_NPs@HG (30 mg/mL)	88.24 ± 0.31

**Table 4:** Co(II) adsorption efficiencies and capacities, obtained from the UV−Vis measurements at the equilibrium, at room temperature.

Adsorbent	Removal% (RE%)	Experimental q_e_ (mg g^−1^)
HG	20.4 ± 0.3	1680 ± 34
γ-Fe_2_O_3_NPs@HG	20.7 ± 0.4	1703 ± 42
γ-Fe_2_O_3_NPs@HG + magnet	25.7 ± 0.6	2111 ± 72

**Table 5:** Co(II) adsorption rate constant obtained from pseudo-first order and pseudo-second order models, at room temperature.

	Pseudo-First Order			Pseudo-Second Order		
Adsorbent	k_1_ (min^−1^)	q_e_ (mg g^−1^)	R^2^	k_2_ (g mg^−1^ min^−1^)	q_e_ (mg g^−1^)	R^2^
HG	0.0036	1867	0.9057	0.0005	1958	0.9722
γ-Fe_2_O_3_NPs@HG	0.0036	1611	0.9363	0.0005	1895	0.9872
γ-Fe_2_O_3_NPs@HG + magnet	0.0032	1486	0.9667	0.0004	2285	0.9834

**Table 6:** Ni(II) adsorption efficiencies and capacities, obtained from the UV−Vis measurements at the equilibrium, at room temperature.

Adsorbent	Removal% (RE%)	Experimental q_e_ (mg g^−1^)
HG	18.6 ± 0.1	1399 ± 12
γ-Fe_2_O_3_NPs@HG	25.9 ± 0.4	1945 ± 41
γ-Fe_2_O_3_NPs@HG + magnet	23.7 ± 0.4	1758 ± 39

**Table 7:** Ni(II) adsorption rate constant obtained from pseudo-first order and pseudo-second order models, at room temperature.

	Pseudo-First Order			Pseudo-Second Order		
Adsorbent	k_1_ (min^−1^)	q_e_ (mg g^−1^)	R^2^	k_2_ (g mg^−1^ min^−1^)	q_e_ (mg g^−1^)	R^2^
HG	0.0115	1948	0.8548	0.000005	1785	0.9735
γ-Fe_2_O_3_NPs@HG	0.0161	2998	0.8247	0.000007	2325	0.9954
γ-Fe_2_O_3_NPs@HG + magnet	0.0092	613	0.9457	0.000036	1851	0.9993

**Table 8:** Cr(III) adsorption efficiencies and capacities, obtained from the UV−Vis measurements at the equilibrium, at room temperature.

Adsorbent	Removal% (RE%)	Experimental q_e_ (mg g^−1^)
HG	13.2 ± 0.1	127 ± 6
γ-Fe_2_O_3_NPs@HG	15.5 ± 0.1	149 ± 8
γ-Fe_2_O_3_NPs@HG + magnet	14.7 ± 0.1	142 ± 5

**Table 9:** Cr(III) adsorption rate constant obtained from pseudo-first-order and pseudo-second-order models, at room temperature.

	Pseudo-First Order			Pseudo-Second Order		
Adsorbent	k_1_ (min^−1^)	q_e_ (mg g^−1^)	R^2^	k_2_ (g mg^−1^ min^−1^)	q_e_ (mg g^−1^)	R^2^
HG	0.0105	248	0.8355	-	27	0.0040
γ-Fe_2_O_3_NPs@HG	0.0101	184	0.9052	0.00003	212	0.9538
γ-Fe_2_O_3_NPs@HG + magnet	0.0112	134	0.9197	0.0001	163	0.9794

## Data Availability

Data available on request.

## Supplementary Materials

The following supporting information can be downloaded at: https://www.mdpi.com/article/10.3390/gels9080621/s1, Figure S1: Separation of γ-Fe_2_O_3_NPs with an external magnetic field (1.42–1.47 T); Figure S2: PAA-coated γ-Fe_2_O_3_NPs solutions after one week for (a) 1.6, 0.16 and 0.08 mg/mL and (b) 10 mg/mL; (c) sedimentation of uncoated γ-Fe_2_O_3_NPs after 24 h.; Figure S3: EDS spectrum of PAA-coated γ-Fe_2_O_3_NPs; Figure S4: UV-Vis study of Co(II) adsorption for (a) the peptide HG; (b) γ-Fe_2_O_3_NPs@HG, and (c) the calibration curve for Co(II) aqueous solutions.; Figure S5: UV-Vis study of Ni(II) adsorption for (a) the peptide HG; (b) γ-Fe_2_O_3_NPs@HG; (c) γ-Fe_2_O_3_NPs@HG upon magnetic field application; (d) the calibration curve for Ni(II) aqueous solutions, and (e) fit of kinetic data to pseudo-first order model for Ni(II).; Figure S6: UV-Vis study of Cr(III) adsorption for (a) the peptide HG; (b) γ-Fe_2_O_3_NPs@HG; (c) γ-Fe_2_O_3_NPs@HG upon magnetic field application, and (d) the calibration curve for Cr(III) aqueous solutions.; Figure S7: Methodology used for studying the removal efficiency of Co(II), as an example here.
