# Supplementary material for: Self-Assembling Peptide-Based Magnetogels for the Removal of Heavy Metals from Water
Source: Gels. 2023 Aug 1;9(8):621. doi: 10.3390/gels9080621 (PMC10454050; doi:10.3390/gels9080621)
Supplement: Supplementary file 1 [file gels-09-00621-s001.zip › gels-2519482-supplementary.pdf]

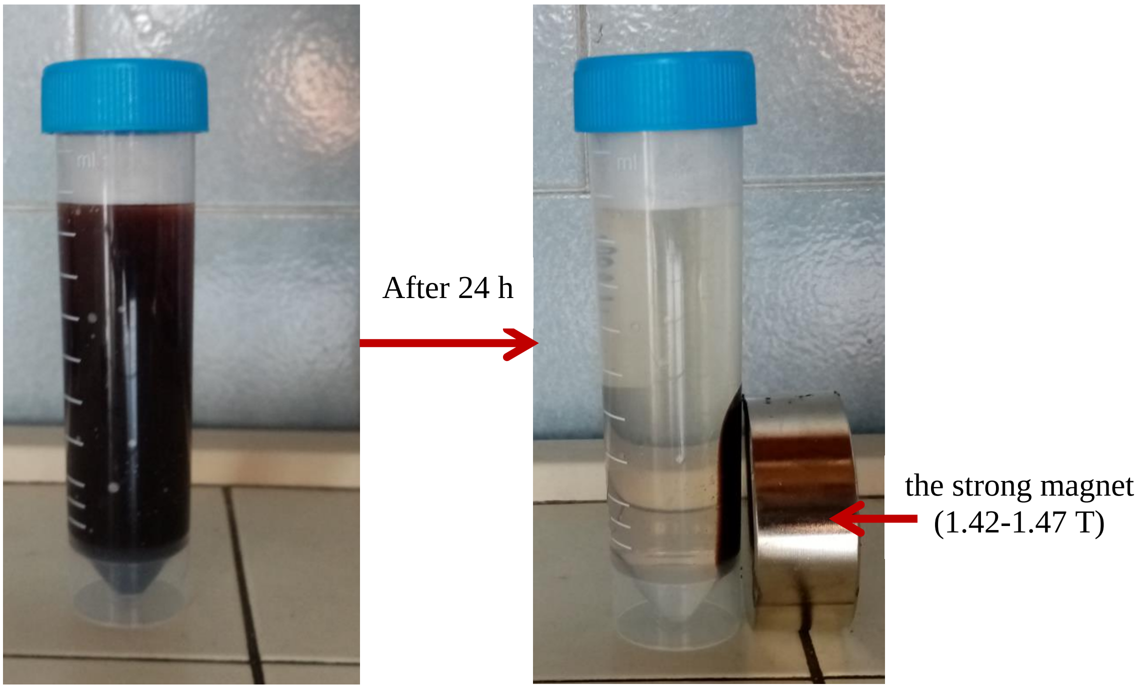

Figure S1. Separation of γ-Fe_2O_3NPs with an external magnetic field (1.42–1.47 T).

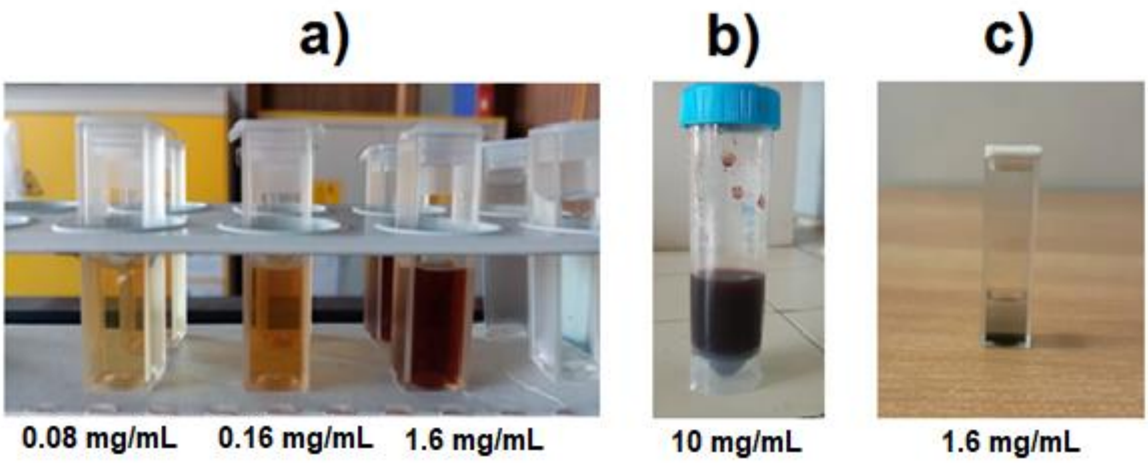

Figure S2. PAA-coated γ-Fe_2O_3NPs solutions after one week for a) 1.6, 0.16 and 0.08 mg/mL and b) 10 mg/mL; c) sedimentation of uncoated γ-Fe_2O_3NPs after 24 h.

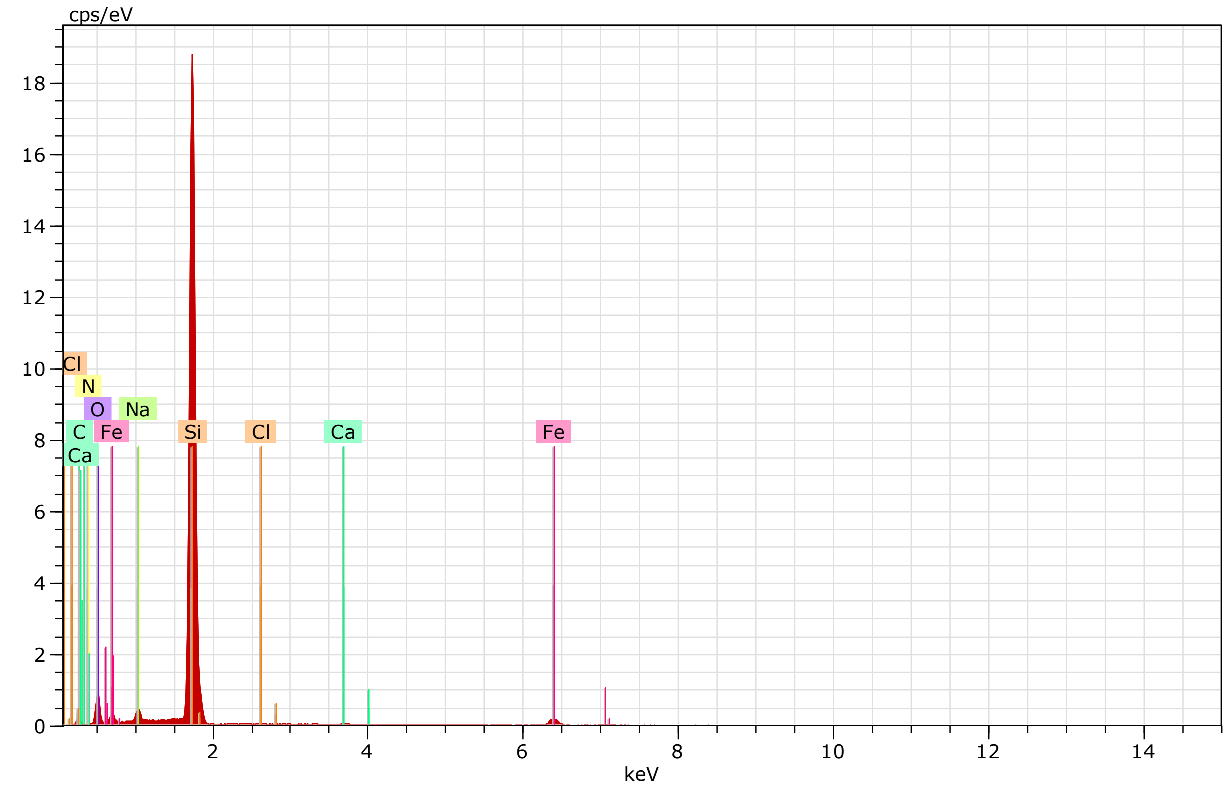

Figure S3. EDS spectrum of PAA-coated γ-Fe_2O_3NPs.

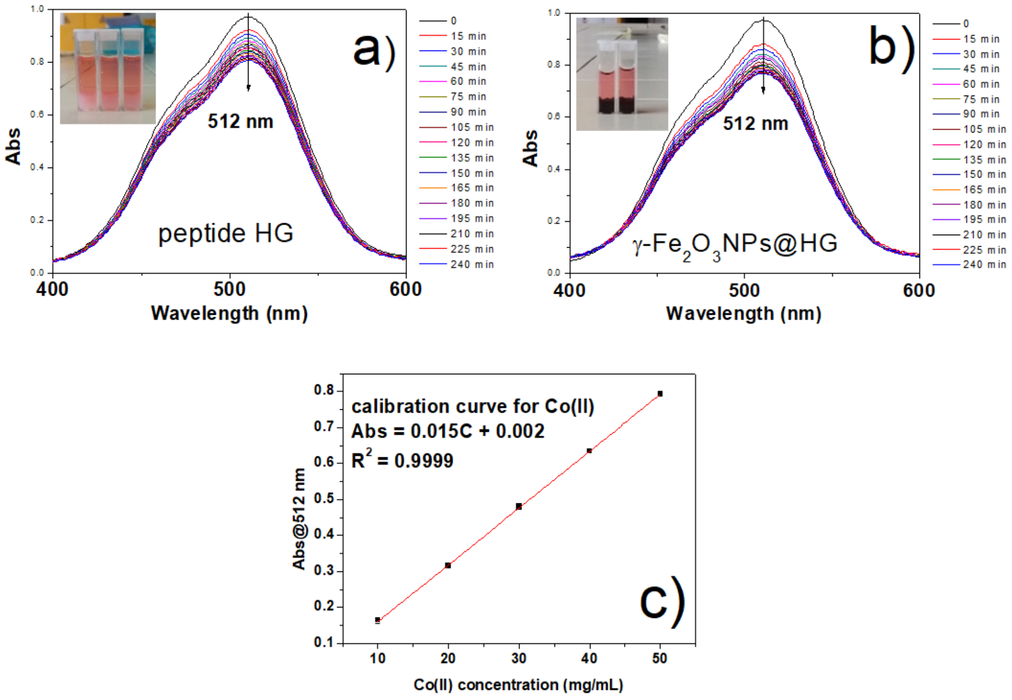

Figure S4. UV-Vis study of Co(II) adsorption for a) the peptide HG; b) γ-Fe_2O_3NPs@HG, and c) the calibration curve for Co(II) aqueous solutions.

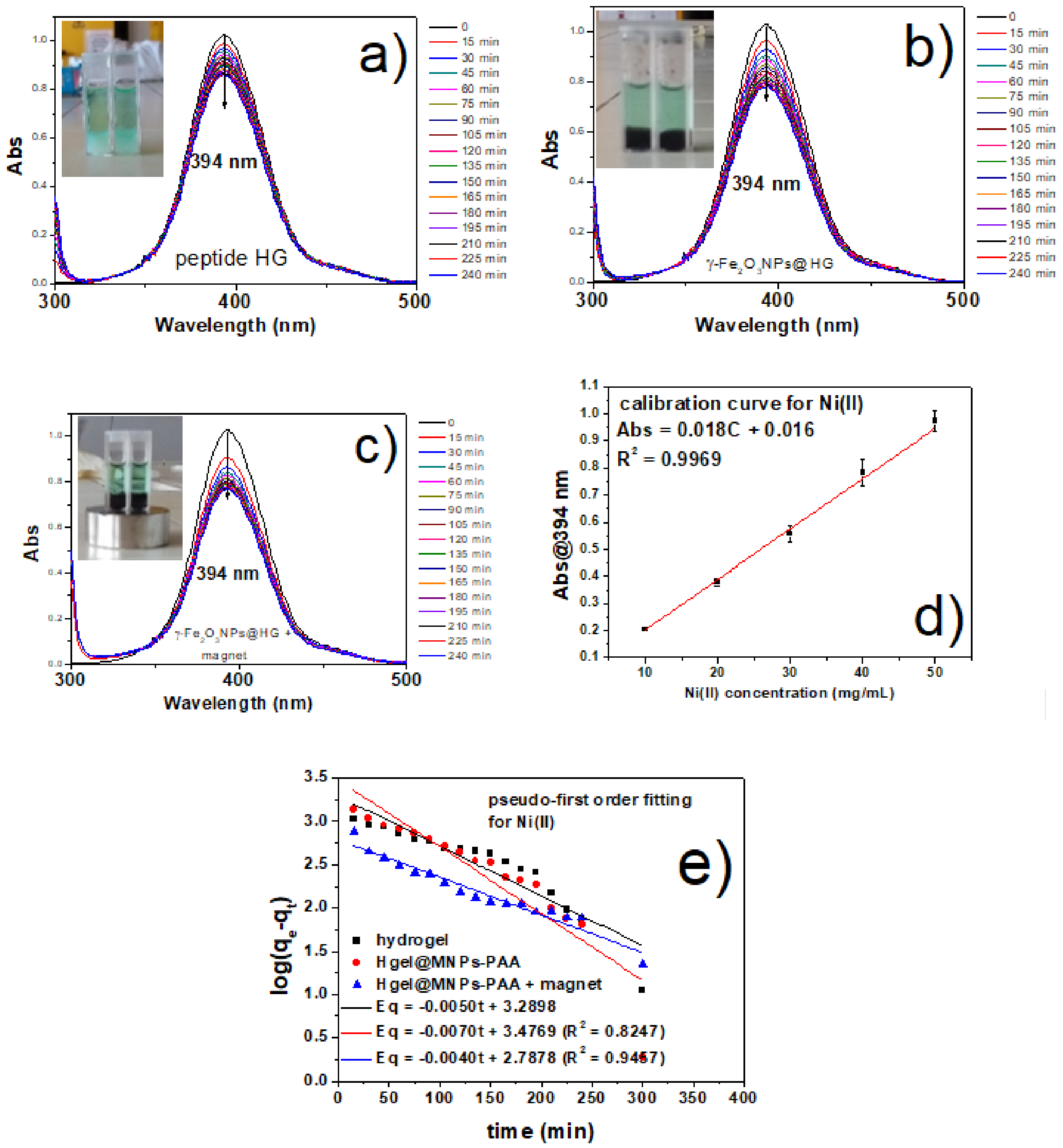

Figure S5. UV-Vis study of Ni(II) adsorption for a) the peptide HG; b) γ-Fe_2O_3NPs@HG; c) γ-Fe_2O_3NPs@HG upon magnetic field application; d) the calibration curve for Ni(II) aqueous solutions, and e) fit of kinetic data to pseudo-first order model for Ni(II).

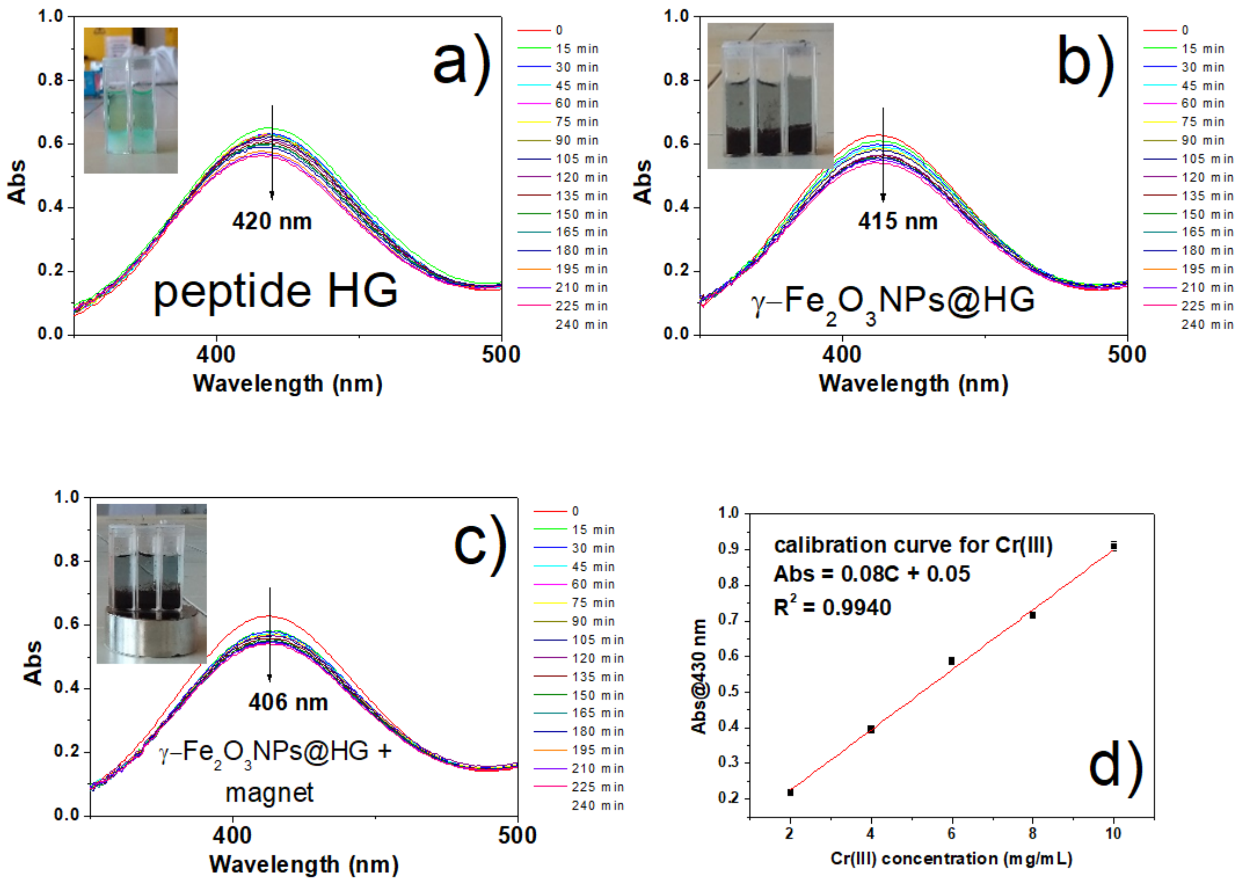

Figure S6. UV-Vis study of Cr(III) adsorption for a) the peptide HG; b) γ-Fe_2O_3NPs@HG; c) γ-Fe_2O_3NPs@HG upon magnetic field application and d) the calibration curve for Cr(III) aqueous solutions.

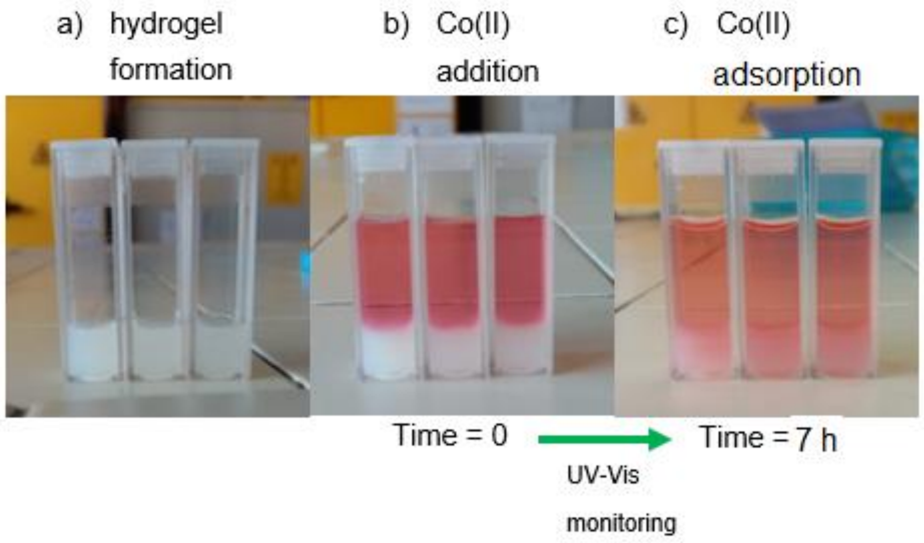

Figure S7. Methodology used for studying the removal efficiency of Co(II), as an example here.
